# The Impact of Chemical Fixation on the Microanatomy of Mouse Organotypic Hippocampal Slices

**DOI:** 10.1523/ENEURO.0104-23.2023

**Published:** 2023-09-25

**Authors:** Agata Idziak, V. V. G. Krishna Inavalli, Stéphane Bancelin, Misa Arizono, U. Valentin Nägerl

**Affiliations:** 1 Unité Mixte de Recherche 5297, Interdisciplinary Institute for Neuroscience, Centre National de la Recherche Scientifique, University of Bordeaux, Bordeaux F-33000, France; 2 Department of Pharmacology, Kyoto University Graduate School of Medicine/The Hakubi Center for Advanced Research, Kyoto University, Kyoto 606-8501, Japan

**Keywords:** dendrites, extracellular space, micro anatomy, mouse brain tissue, PFA fixation artefacts, STED microscopy

## Abstract

Chemical fixation using paraformaldehyde (PFA) is a standard step for preserving cells and tissues for subsequent microscopic analyses such as immunofluorescence or electron microscopy (EM). However, chemical fixation may introduce physical alterations in the spatial arrangement of cellular proteins, organelles, and membranes. With the increasing use of super-resolution microscopy to visualize cellular structures with nanometric precision, assessing potential artifacts, and knowing how to avoid them, takes on special urgency. We addressed this issue by taking advantage of live-cell super-resolution microscopy that makes it possible to directly observe the acute effects of PFA on organotypic hippocampal brain slices, allowing us to compare tissue integrity in a “before-and-after” experiment. We applied super-resolution shadow imaging (SUSHI) to assess the structure of the extracellular space (ECS) and regular super-resolution microscopy of fluorescently labeled neurons and astrocytes to quantify key neuroanatomical parameters. While the ECS volume fraction (VF) and microanatomic organization of astrocytes remained largely unaffected by the PFA treatment, we detected subtle changes in dendritic spine morphology and observed substantial damage to cell membranes. Our experiments show that PFA application via immersion does not cause a noticeable shrinkage of the ECS in hippocampal brain slices maintained in culture, unlike the situation in transcardially perfused animals *in vivo* where the ECS typically becomes nearly depleted. Our study outlines an experimental strategy to evaluate the quality and pitfalls of various fixation protocols for the molecular and morphologic preservation of cells and tissues.

## Significance Statement

Chemical fixation of biological samples using paraformaldehyde (PFA) is a standard step routinely performed in neuroscience labs. However, it is known to alter various anatomic parameters ranging from protein distribution to cell morphology, potentially affecting our interpretation of anatomic data. With the increasing use of super-resolution microscopy, understanding the extent and nature of fixation artifacts is an urgent concern. Here, we use live stimulated emission depletion (STED) microscopy to monitor in real time the impact of PFA on the microanatomy of organotypic hippocampal brain slices. Our results demonstrate that while PFA has little impact on the extracellular space (ECS) and astrocytes, it compromises cell membranes and dendritic structures. Our study provides a strategy for a direct characterization of fixation artifacts at the nanoscale, facilitating the optimization of fixation protocols.

## Introduction

Chemical fixation is a commonly used preservation step for electron microscopy (EM) and super-resolution microscopy techniques, such as stimulated emission depletion (STED) microscopy, single-molecule localization microscopy and expansion microscopy. These techniques permit structural and molecular analyses of cells and tissues at a submicroscopic level. Chemical fixatives like paraformaldehyde (PFA) covalently cross-link proteins, which has the effect of physically hardening the cellular and molecular structure of the sample (Fox et al., 1985). This procedure is to protect the sample from decay and damage during subsequent processing steps, such as tissue slicing, dehydration or embedding in resin.

However, it is known that even the most carefully executed fixation protocol can introduce structural artifacts that compromise data quality and interpretation (Ebersold et al., 1981; Ryter, 1988; Wyffels, 2001; Schnell et al., 2012). While these problems are perhaps not noticeable at a macroscopic level, they might appear at the microscopic subcellular scale. Indeed, organelles such as endosomes and lysosomes become deformed by chemical fixation (Murk et al., 2003), while cellular proteins can still move substantially and reposition after chemical fixation, potentially casting doubts over conclusions based on this approach (Tanaka et al., 2010).

As the spatial resolution of modern microscopy techniques keeps improving, allowing researchers to make ever more detailed and discriminating observations, concerns about fixation artifacts become more relevant. Recent super-resolution techniques can now reach into the low nanometer range (Arizono et al., 2023), where fixation artifacts may abound. In turn, these gains in spatial resolution necessitate the development of more stringent ways to assess the quality of fixation protocols and how well they can preserve cellular elements at this finer spatial scale.

The question of how much the microarchitecture and ultrastructure of brain tissue is affected by chemical fixation was addressed in two EM studies that compared the effects of chemical and cryogenic fixation protocols on tissue fine structure. Cryogenic fixation is based on rapid high-pressure freezing of the sample, which produces amorphous ice instead of ice crystals, that otherwise would destroy the ultrastructure. These studies clearly showed that chemical fixation via transcardial perfusion leads to a strong depletion of the extracellular space (ECS) as well as changes in astrocytic (Korogod et al., 2015) and dendritic spine morphology (Tamada et al., 2020), raising serious concerns about the use of chemical fixation protocols in high-resolution anatomic studies of brain tissue. However, because of differences in sample preparation required for either fixation method (transcardial perfusion of the brain *in vivo* with buffered PFA versus rapid cryo-genic freezing of acute brain slices with subsequent storage in liquid nitrogen) and the inability to compare the EM samples with their live originals, the reason of the observed differences remains elusive.

To directly compare nanoscale neuroanatomical structures before and after chemical fixation, we took advantage of the super-resolution shadow imaging (SUSHI) technique, which is based on 3D-STED microscopy and fluorescence labeling of the interstitial fluid (Tønnesen et al., 2018). SUSHI allows for visualization of tissue anatomy, including the ECS, projecting all cellular structures as sharply contoured “shadows,” providing a comprehensive and nonbiased view of the cellular architecture of the tissue. Using this technique, we imaged organotypic brain slices and analyzed the impact of PFA on the ECS. In addition, we imaged fluorescently labeled astrocytes and neurons, and analyzed the effect of PFA on their nanoscale morphology in a before-and-after manner.

We observed that PFA does not induce major changes in the shape and size of the ECS and astrocytes. However, we detected subtle changes in dendritic spine morphology as well as a widespread disruption of cellular membranes and cellular blebbing.

The study gives a “real time” and nanoscale view of the effects of PFA on tissue microarchitecture of cultured hippocampal slices, revealing the extent and type of fixation artifacts, which had remained inconclusive. Applying this approach to other brain areas and tissue preparations (acute brain slices, intact brain *in vivo*) may help the development of fixation protocols to preserve the native structure of the tissue as well as possible.

## Materials and Methods

### Mouse line

Animal experimental procedures were in accordance with the French National Code of Ethics on Animal Experimentation and approved by the Committee of Ethics of Bordeaux. All procedures were performed according to the guidelines of the European Directive 2010/63/UE.

Mice were housed under a 12/12 h light/dark cycle cycle at 20–22°C with *ad libitum* access to food and water in the animal facility of the Interdisciplinary Institute for Neuroscience (University of Bordeaux/Centre National de la Recherche Scientifique) and monitored daily by trained staff. All animals used were free of any disease or infection at the time of experiments. Pregnant females and females with litters were kept in cages with one male. We did not distinguish between males and females among the perinatal pups used for organotypic cultures, as potential anatomic and/or physiological differences between the two sexes were considered irrelevant in the context of this study.

C57Bl/6J wild-type mice were used for all experiments in this study.

### Organotypic brain slices

Organotypic hippocampal slices (Gähwiler, 1981) were dissected from 5- to 7-d-old mice, and were cultured for two to five weeks in a roller drum at 35°C (for details, see Tønnesen et al., 2018). Once a week, 500 μl of medium was exchanged in the tubes. For experiments, a given coverslip with a slice was mounted in an imaging chamber, and the slice was imaged from below through the glass coverslip, while PFA-containing solutions were added to the imaging chamber from above.

### Viral injections

In order to fluorescently label neurons or astrocytes, we have introduced either Sindbis-Citrine or AAV2/1.gfaABC1D-Clover viruses to the brain slices via microinjections using a glass pipette connected to a Picospritzer (Parker Hannifin). Briefly, the virus was injected via a pipette positioned into the CA1 area of the slice by brief pressure pulses (30 ms; 15 psi). For imaging of the neurons, Sindbis-Citrine virus was injected into two-week-old wild-type slices 1 d before the experiments. To image astrocytes, two-week-old wild-type slices were injected with AAV2/1.gfaABC1D-Clover two weeks before the experiments.

### Extracellular labeling

Extracellular labeling of organotypic slices was performed as described before (Tønnesen et al., 2018). In brief, once the slice was transferred to the imaging chamber, it was immersed in HEPES-buffered artificial CSF (ACSF), containing 200 μm of the fluorescent dye Calcein (Dojindo Laboratories).

### Chemical fixation

After acquiring a live image of a slice (where either the ECS, neurons or astrocytes were labeled), the Calcein/ACSF or ACSF-only solutions were carefully removed with a pipette, making sure to keep drift of the slice to a minimum. Subsequently, solutions containing 4% PFA diluted in HEPES-based ACSF with or without Calcein were pipetted on top of the slice. To minimize the evaporation of PFA, the imaging chamber was covered with a lid.

For overnight chemical fixation, the organotypic slices on a glass coverslip were transferred from the roller drum tube to a 6-well plate and instantly immersed in 4% PFA diluted in 1× PBS solution. The 6-well plate was placed at 4°C overnight, followed by three washes in PBS. Finally, the fixed slices on the glass coverslip were mounted onto the imaging chamber containing Calcein/ACSF solution.

### 3D-STED microscopy

We used a home-built 3D-STED setup (for details, see Inavalli et al., 2019) constructed around an inverted microscope body (DMI 6000 CS, Leica Microsystems), which was equipped with a TIRF oil objective (100×, 1.47 NA, HXC APO, Leica Microsystems) and contained within a heating box (Cube and Box, Life Imaging Services) to maintain a stable temperature of 32°C. A pulsed-laser (PDL 800-D, PicoQuant) was used to deliver excitation pulses (90 ps at 80 MHz) at 485 nm and a synchronized de-excitation laser (Onefive Katana 06 HP, NKT Photonics) operating at 592 nm was used to generate the STED light pulses (500–700 ps). The STED beam was reflected on a spatial light modulator (SLM, Easy3D Module, Abberior Instruments) to generate a mixture of doughnut-shaped and bottle-shaped beams for 2D and 3D-STED, respectively. Image acquisition was controlled by the Imspector software (Abberior Instruments). The performance and spatial resolution of the microscope was checked and optimized by visualizing and overlapping the PSFs of the laser beams using 150-nm gold nano-spheres and 40-nm fluorescent beads and correcting the main optical aberrations with the SLM. The spatial resolution was 175 nm (lateral) and 450 nm (axial) in confocal mode and 60 nm (lateral) and 160 nm (axial) in STED mode.

### Image acquisition

For imaging, slices were transferred on their glass coverslip to the imaging chamber and immersed in ACSF consisting of the following (in mm): 119 NaCl, 2.5 KCl, 1.3 MgSO_4_, 1 NaH_2_PO_4_ × 2H_2_O, 2.5 CaCl_2_ × 2H_2_O, 20 D-glucose × H_2_O, and 10 HEPES (all from Sigma-Aldrich); 300 mOsm; pH 7.4. We acquired confocal image stacks with the following parameters: 100 × 100 × 4 μm^3^ with a Δz size of 1 μm and a pixel size of 48.8 nm. For STED imaging, we either acquired 100 × 100 μm^2^ single sections or z-stacks (15 × 15 × 1 or 25 × 25 × 1 μm^3^ with a Δz size of 200 nm). All STED images were acquired with a pixel size of 19.53 nm and a pixel dwell time of 30 μs. The excitation power was 0.5 μW and STED power was 30 mW at the entrance pupil of the objective. We adjusted the *z* focus and the *xy* stage to keep sample drift to a minimum between image acquisitions. Images were cropped at the edges if needed to keep the field of view the same.

### Image processing and analysis

#### ECS volume fraction and widths

**Figure 1.**
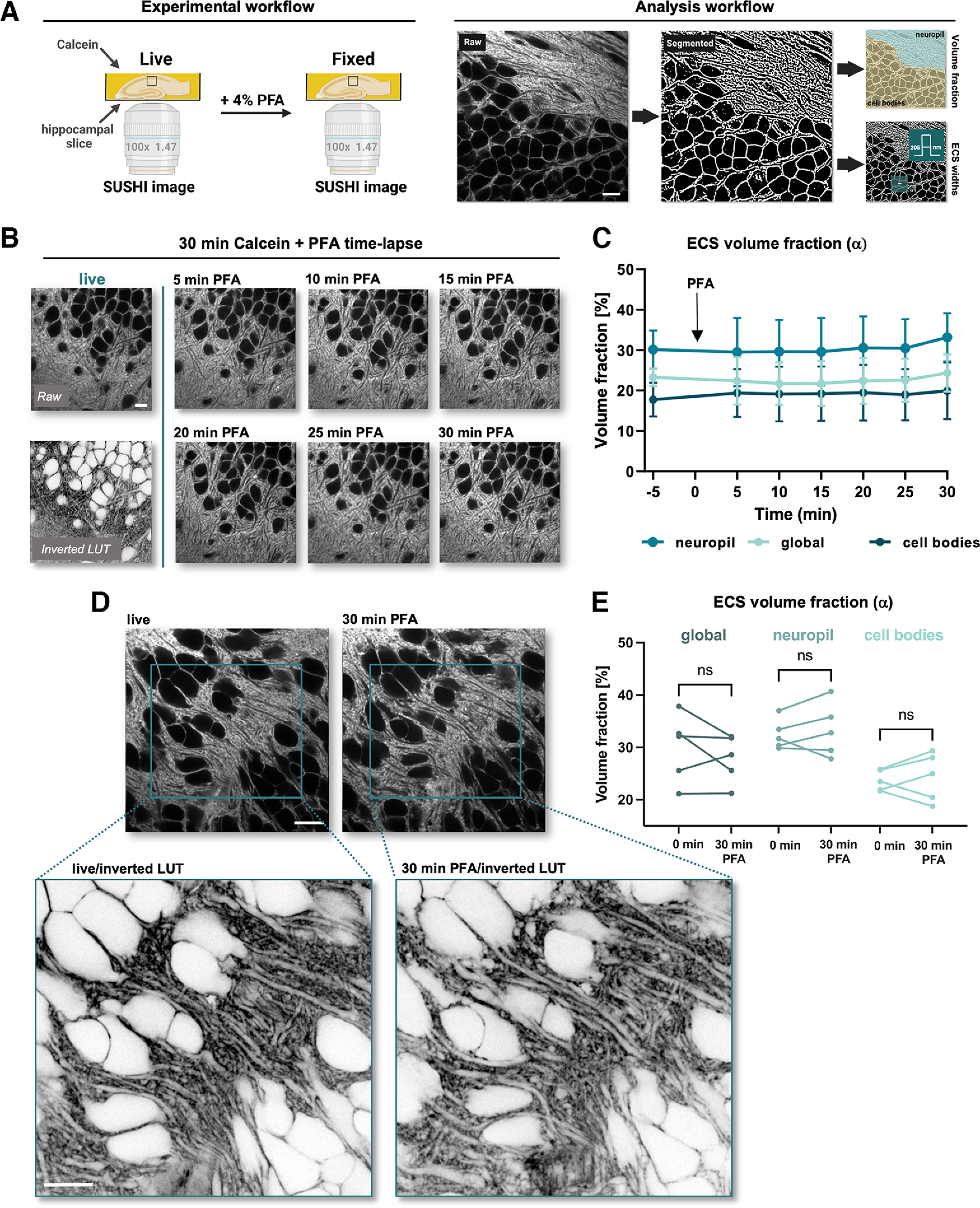
Brief PFA incubation does not affect ECS volume fraction. ***A***, Graphical overview of the workflow of experiments and analysis. ***B***, Time-lapse shadow imaging of ECS in live and buffered PFA conditions. The live condition is represented both with a raw and inverted LUT. ***C***, ECS volume fraction α does not change during a 30-min incubation with PFA. The images were analyzed either as a whole (“global”) or divided into “neuropil” or “cell bodies” areas (*n* = 6; presented as mean + SD). ***D***, Representative images of ECS live and after 30 min of incubation with PFA. Blue squares indicate the magnified area that is shown below with an inverted LUT. ***E***, Paired analysis of ECS volume fraction live and after 30 min of incubation with PFA (*n* = 5; ns: not significant; *p* > 0.05; in paired *t*-test). Scale bars: 10 μm.

SUSHI images are single sections taken from z-stacks or time-lapse series for Figure 1*B*, selected to match the *x-y-z* planes. Brightness and contrast were adjusted for each individual image using ImageJ (NIH) and the look-up table (LUT) was “grays.” To measure the ECS volume fraction (VF) and widths, images were binarized using a wavelet-based software SpineJ (Levet et al., 2020; Fig. 1*A*). For the VF (α), we computed the ratio of pixels representing ECS over the total number of pixels (α = *N*_ECS_/*N*_total_) in a region of interest. For ECS widths, we analyzed three-pixel-wide line profiles drawn across the binarized images.

#### Measurements of astrocytic structures and dendritic spines

Images of astrocytes and dendrites are shown as maximum intensity z-projections with the LUT “orange hot.” Areas of astrocytic cell bodies and main branches were measured by drawing a contour line around the structures and calculating their surface areas in ImageJ. The widths of astrocytic fine processes were measured on raw images in ImageJ, using the “Plot Line Profile” function after drawing a 3-pixel-wide straight line across the structure of interest. “Dendritic vacuoles” were visually identified and quantified by counting them in the images acquired before and after PFA application. Morphologic parameters of dendritic spines were measured using the SpineJ software. The software identifies the neck region and places lines that are orthogonal to the neck axis at regular intervals of 75 nm. It calculates the full-width at half-maximum (FWHM) of the neck diameter, returning the minimum, maximum and median values for each analyzed spine. We limited the morphology analysis to dendritic spines with clear neck and head compartments, commonly referred to as mushroom spines.

### Experimental design and statistical analysis

Statistical tests were performed using GraphPad Prism software. Normality tests were performed to confirm data were normally distributed. Paired *t* tests were performed for normally distributed data and Wilcoxon or Mann-Whitney tests for non-normally distributed data. The size and type of individual samples, n, for given experiments is indicated and specified in Results and figure legends. Asterisks in figures indicate *p* values as follows: *p < 0.05, **p < 0.01, ***p < 0.001, ****p < 0.0001. Image analysis and statistical tests were performed blind to the experimental condition.

## Results

### Thirty minutes of PFA fixation has no detectable effects on ECS volume fraction

To assess whether chemical fixation using PFA has an effect on hippocampal ECS structure, we established an experimental workflow that allowed us to compare the same sample before and after PFA fixation in a paired manner (Fig. 1*A*). As a readout of the ECS structure, we analyzed the volume fraction (α) and geometric widths of the ECS. The volume fraction α is an important structural parameter of brain tissue (Nicholson and Hrabětová, 2017), which is defined by the ratio of the ECS volume over the total volume in a region of interest.

We performed time-lapse confocal shadow imaging at 5-min intervals before and during PFA application using Calcein to label the ACSF that the slices were maintained in Figure 1*B*. The images were binarized using SpineJ software (Levet et al., 2020) based on wavelet filtering, allowing us to calculate the ratio of the number of “ECS pixels” over the total number of pixels as an estimate of the volume fraction in a region of interest. We found that 30 min of PFA incubation did not cause any significant changes in the ECS volume fraction (Fig. 1*C*; *n*_PFA_ = 6; *p* = 0.3962; paired *t* test).

This result was confirmed by shadow-imaging with 3D-STED (SUSHI; Fig. 1*D*,*E*; *n* = 5; *p* = 0.3066; paired *t* test), indicating that 30 min of PFA incubation has little impact on the volume fraction of the ECS in organotypic hippocampal slices.

### Prolonged PFA incubation introduces pronounced artifacts

**Figure 2.**
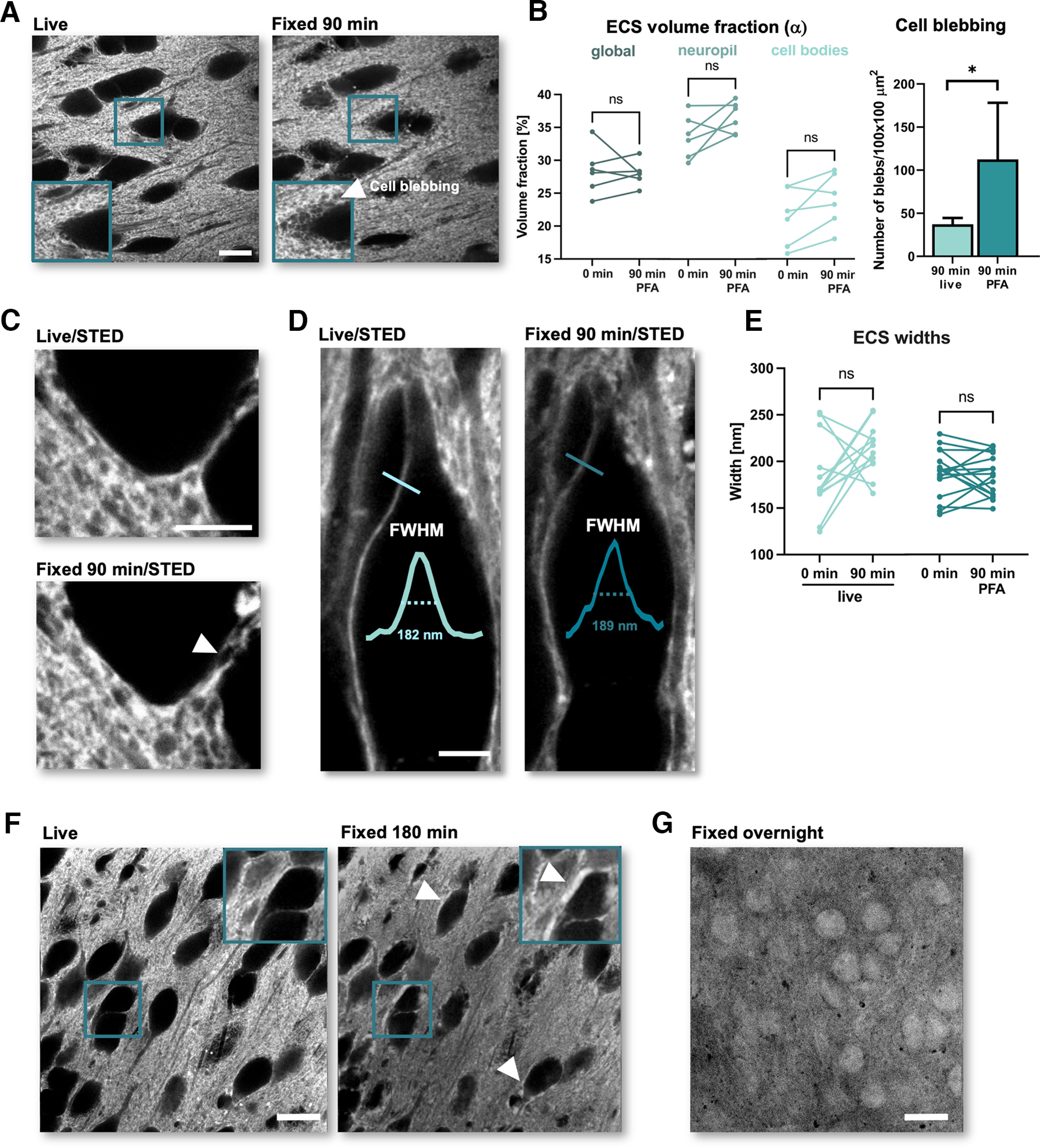
Prolonged PFA incubation introduces artifacts into ECS structure. ***A***, Representative images of ECS live and after 90 min of incubation with PFA. The inset and white arrow indicate “cell blebbing.” ***B***, Left, Paired analysis of ECS volume fraction between live and 90 min after PFA fixation. The images were analyzed either as a whole (“global”) or divided into “neuropil” or “cell bodies” areas (*n*_cntrl_ = 6; *n*_PFA_ = 6; ns: not significant; *p* > 0.05; in Wilcoxon matched-pairs test). Right, Comparison of the “cell blebbing” between live and 90-min PFA conditions (*n* = 6; *p < 0.05; Mann–Whitney test). ***C***, Representative STED images of ECS live and after 90 min of PFA fixation. ***D***, SUSHI-based analysis of ECS widths. The blue lines indicate an example of the analyzed width. The line profiles are shown together with measured FWHMs. ***E***, Paired analysis of ECS widths live and 90 min after PFA fixation (*n*_cntrl_ = 12; *n*_PFA_ = 16; ns: not significant; *p* > 0.05; paired *t* test). ***F***, Representative images of ECS live and 180 min after PFA fixation. Inset and white arrows indicate examples of dye accumulation around cell bodies. ***G***, A representative confocal shadow image after fixation overnight with PFA. Scale bars (***A***, ***F***, ***G***): 10 μm; (***C***, ***D***): 5 μm.

As brain slices are often maintained in fixative for more than 1 h or even overnight, we investigated the effects of longer incubation times on ECS structure (Fig. 2*A*). We maintained the slices for 90 min under PFA or normal ACSF conditions and compared SUSHI images before and after this period. Even 90 min of PFA fixation appeared to affect neither ECS volume fraction (Fig. 2*B*, left; *n* = 6; *p*> 0.05; Wilcoxon matched-pairs test) nor widths measured in line profiles of binarized images using SpineJ (Fig. 2*C–E*; *n*_cntrl_ = 12 lines, *n*_PFA_ = 16 lines; *p*_cntrl_ = 0.1281; *p*_PFA_ = 0.7249; paired *t* test).

However, after 90 min under PFA conditions we observed dye-free, cellular blebs in the immediate vicinity of cell bodies (Fig. 2*A*,*C*, white arrows; Fig. 2*B*, right, *n*_live_ = 6, *n*_PFA_ = 6; *p* = 0.0043; Mann–Whitney test).

Incubation for 180 min with PFA caused even more pronounced changes, such as dye accumulation around cell bodies and dye permeation into the cells (Fig. 2*F*), indicating that PFA by itself permeabilized cell membranes, even in the absence of detergents, like Triton, that are typically used in immunofluorescence protocols for antibodies to reach intracellular epitopes (Goldenthal et al., 1985). Indeed, after overnight PFA incubation the extracellular dye had entered into the cells (Fig. 2*G*), indicating disruption of cellular membranes. This made assessing the impact of PFA on the ECS impossible, because the inside-outside contrast, which is required for the shadow imaging approach, was lost. Thus, >90 min of PFA fixation appears to seriously damage the integrity of cellular membranes.

### Ninety minutes of PFA fixation does not affect the morphology of astrocytes

**Figure 3.**
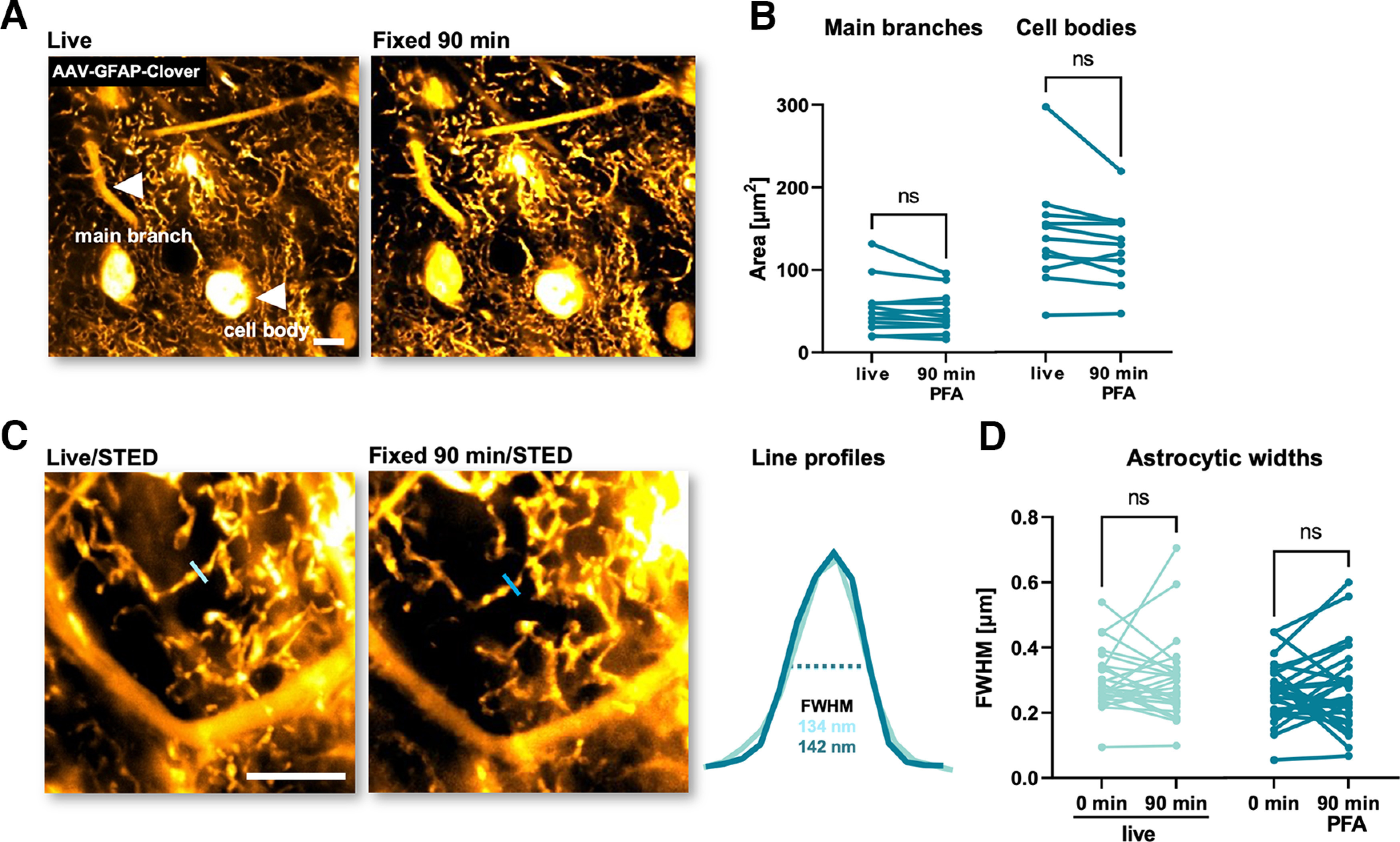
PFA fixation has no detectable effects on astrocytic fine structure. ***A***, Representative confocal images of a brain slice expressing GFAP-Clover in astrocytes, live and 90 min after PFA fixation. White arrows indicate a representative astrocytic main branch and cell body. ***B***, Paired analysis of astrocytic areas of main branches and cell bodies live and 90 min after PFA fixation (*n*_branches_ = 12; *n*_bodies_ = 11; ns: not significant; *p* > 0.05; paired *t* test). ***C***, Representative STED images of astrocytic structures in spongiform domain expressing GFAP-Clover, live and 90 min after PFA fixation. The blue lines show representative line profiles of astrocytic structures to determine their width. The line profiles are shown on the right together with calculated FWHMs. ***D***, Paired analysis of widths of astrocytic fine processes, live and 90 min after PFA fixation (*n*_cntrl_ = 26; *n*_PFA_ = 28; ns: not significant; *p* > 0.05; paired *t* test). Scale bars: 10 μm.

In addition to the effects of PFA on ECS structure, we set out to determine the impact of PFA fixation on different cell types. We first focused on astrocytes, whose morphology is known to be very sensitive to environmental changes, such as osmotic challenges (Arizono et al., 2021) and transcardial perfusion of fixatives (Korogod et al., 2015). In order to label astrocytes, we microinjected AAV-GFAP-Clover viral particles into organotypic hippocampal slices. Confocal microscopy did not turn up any significant changes in the size of the major branches and cell bodies of astrocytes after 90 min of PFA fixation (Fig. 3*A*,*B*; *n*_branches_ = 12; *n*_bodies_ = 11; *p*_branches_ = 0.0952; *p*_bodies_ = 0.1322; paired *t* test). Likewise, STED microscopy did not detect any significant changes in the widths of astrocytic fine processes (Fig. 3*C*,*D*; *n*_cntrl_ = 26; *n*_PFA_ = 28; *p*_cntrl_ = 0.9589, *p*_PFA_ = 0.9022; paired *t* test). These results suggest that PFA incubation by itself has surprisingly little impact on astrocytic morphology.

### Ninety minutes of PFA fixation leads to changes in dendritic spine morphology

**Figure 4.**
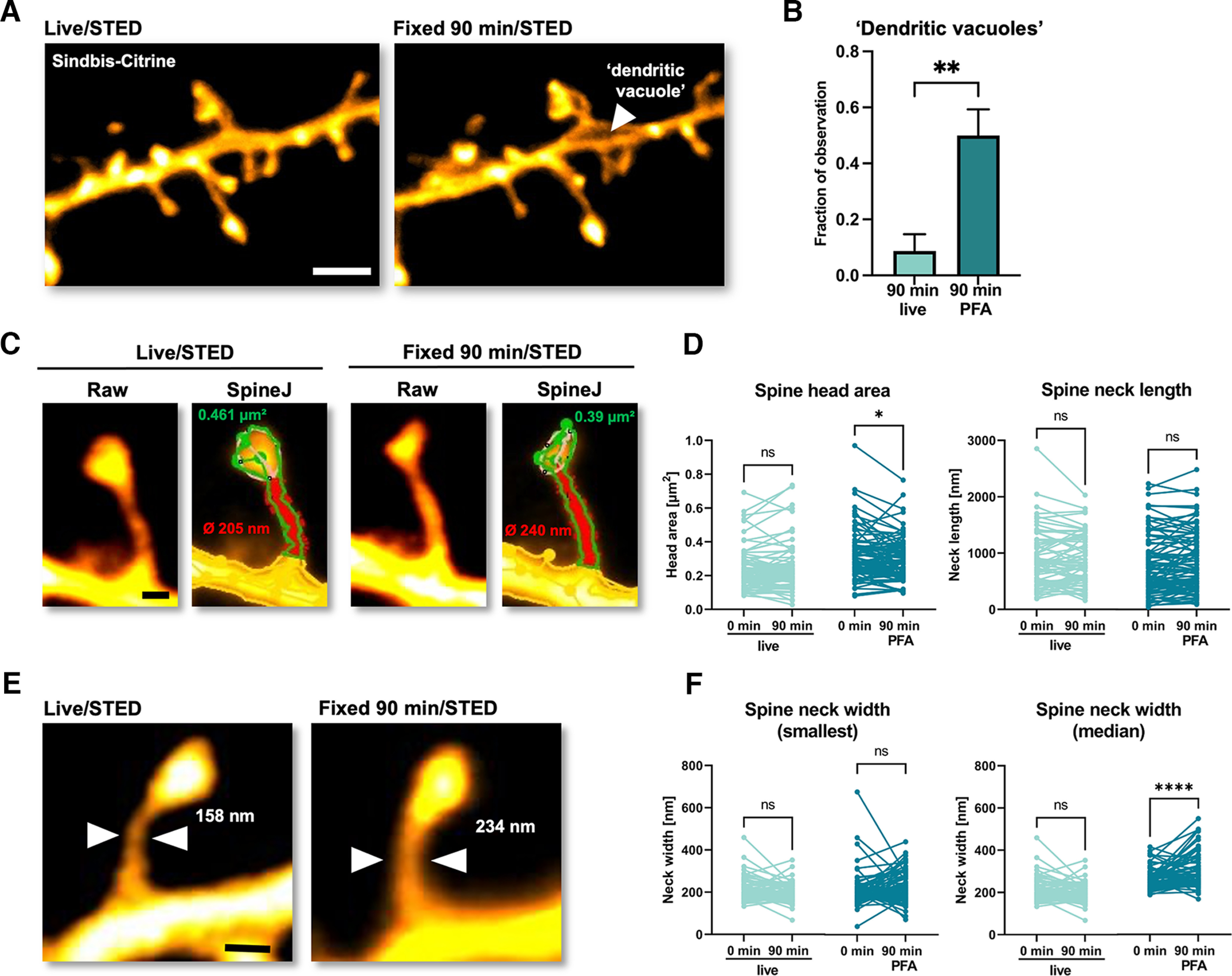
PFA fixation affects spine morphology. ***A***, Representative STED maximum intensity z-projections of a dendritic segment cytosolically filled with Citrine, live and 90 min after PFA fixation. White arrow indicates a “dendritic vacuole.” ***B***, Bar graph showing appearance of “dendritic vacuoles” after 90 min under live or fixed conditions (expressed as appearances over total number of experiments: control: 2 out of 23; PFA: 15 out of 31; *p* = 0.0024; bars show mean + SD; Mann–Whitney test). ***C***, Representative STED images of dendritic spines and an example of head and neck analysis using SpineJ. ***D***, Paired analysis of spine head area and neck length (*n*_cntrl_ = 79; *n*_PFA_ = 86; ns: not significant; *p* > 0.05; *p = 0.0157; Wilcoxon matched-pairs test). ***E***, An example of a dendritic spine with a wider neck after 90 min of PFA fixation. ***F***, Paired analysis of spine neck width (smallest and median values) between live and 90-min PFA-fixed conditions (*n*_cntrl_ = 79; *n*_PFA_ = 86; ns: not significant; ****p < 0.0001; Wilcoxon matched-pairs test). Scale bars (***A***): 10 μm; (***C***, ***E***): 500 nm.

Finally, we performed similar experiments with neurons by virally labeling them with Citrine as fluorescent protein. Using STED microscopy, we imaged dendrites and dendritic spines before and after 90 min of PFA fixation (Fig. 4*A*). Unlike astrocytes, many neurons formed “dendritic vacuoles” after exposure for 90 min to PFA, which could be extracellular holes in the dendrites or intracellular vacuoles free of the fluorescent label. As we did not observe any dye-filled holes in dendrites in SUSHI images, these structures most likely reflect Citrine-free spaces formed by coalescing intracellular organelles such as mitochondria. In control experiments, i.e., in the absence of PFA, we rarely observed such structures (Fig. 4*B*; *n*_cntrl_ = 23, *n*_PFA_ = 31; *n* = number of analyzed dendritic segments; *p* = 0.0074; Mann–Whitney test).

Quantitative analysis of dendritic spine morphology revealed that there were no significant changes in spine neck lengths (Fig. 4*D*; *p* length = 0.6507; Wilcoxon matched-pair test); however, spine head areas became significantly smaller (Fig. 4*C*,*D*) and the median spine neck diameter (measured along the length of the neck; for details, see Materials and Methods) became wider after 90 min of PFA treatment (Fig. 4*E*,*F*; *p*_h.area_ = 0.0157; *p*_width_ < 0.0001; Wilcoxon matched-pair test). While this effect was statistically highly significant for the median neck diameter, it was not for the thinnest parts of the spine necks (Fig. 4*F*; *p*_width_ = 0.1565; Wilcoxon matched-pair test).

These results show that dendrites and spines are affected by PFA fixation, suggesting that neurons are more sensitive than astrocytes to PFA treatment in organotypic brain slices within the limits of the resolution of our STED imaging approach.

## Discussion

In this study, we describe the impact of chemical fixation with buffered PFA on tissue architecture in organotypic hippocampal slices, focusing on the ECS and cellular morphology. Our SUSHI approach indicates that immersion of organotypic brain slices in PFA up to 90 min does not affect ECS volume fraction and widths. Similarly, STED imaging showed that astrocytic morphology remains unaffected by PFA even at the level of the fine processes. However, subtle changes in spine morphology appeared within these relatively brief applications of PFA (Table 1).

Previous studies have shown that transcardial perfusion of PFA reduces ECS widths (Thorne and Nicholson, 2006) and causes major ECS shrinkage and astrocytic swelling (Korogod et al., 2015). The fact that our experiments did not reveal such changes indicates that they were not a direct result of PFA by itself on the tissue, but rather a consequence of the anomalies subsequent to transcardial PFA perfusion, such as anoxia (Tao-Cheng et al., 2007), or dehydration. This is in line with the observation that acute slices fixed either chemically or cryogenically did not appear different in terms of tissue quality and ECS distribution (Korogod et al., 2015). Given the absence of major remodeling of the ECS under our conditions, it is perhaps not surprising that the astrocytes did not show any changes either, suggesting that their morphology and ECS topology are closely linked (Korogod et al., 2015).

Transcardial fixation was also shown to impact the spatial and molecular organization of synaptic vesicles (Maus et al., 2020). In the same vein, a recent study showed that PFA affects the behavior of proteins in liquid–liquid phase separation experiments, underscoring the importance of understanding better the artifacts induced by PFA fixation (Irgen-Gioro et al., 2022).

PFA fixation over 90 min considerably disturbed membrane integrity as indicated by the penetration of the extracellular dye into the cells, preventing us from acquiring SUSHI images. The membrane permeabilization was accompanied with cellular blebbing, which has already been observed in cell cultures (Zhao et al., 2014), suggesting that this is a common effect of PFA. Immunofluorescence experiments with and without membrane permeabilization showed that PFA permeabilizes the membrane to an extent that an antibody can pass into the cells (data not shown). Such loss of membrane integrity needs to be considered in experiments focused on membrane proteins, including ion channels or surface receptors (Ichikawa et al., 2022), as well as many intracellular proteins that are linked to these proteins, such as components of the postsynaptic density (Chen et al., 2008). A loss of membrane integrity may thus distort our view of the synapse.

Ninety minutes of PFA fixation also induced “dendritic vacuoles,” which were virtually absent in control experiments. Morphometric analysis of dendritic spines revealed that PFA application leads to wider spine necks, in line with previous findings, where spine necks were 30% thinner in cryogenically than chemically fixed samples (Tamada et al., 2020). While we did not see any changes in spine neck length, we observed slightly decreased spine head sizes.

To summarize, the time-lapse super-resolution approach allowed us to compare the same tissue sample at the nanoscale under live and fixed conditions. Whereas short-lasting fixation (<30 min) is largely innocuous to tissue structure, longer PFA applications unmistakably lead to artifacts. However, as our study was conducted in an artificial system (organotypic brain slices), we do not know what happens *in vivo*, where other factors (e.g., anoxia) come into play. In principle, STED imaging *in vivo* (Pfeiffer et al., 2018) during transcardial perfusion of fixatives could address this knowledge gap.

With the proliferation of super-resolution studies based on chemical fixation, it is crucial to reveal the effects of chemical fixatives, ambient conditions (e.g., temperature) and other sample preparation steps on the native molecular and structural organization of the samples, to optimize fixation protocols (Whelan and Bell, 2015; Pereira et al., 2019; Laporte et al., 2022). Single-molecule imaging, ideally in combination with STED (Inavalli et al., 2019), could provide a very sensitive readout to check protein arrangements and cell morphology in parallel, helping to determine effective and practical solutions to increase the preservation of fixed cells and tissues and the fidelity of their microscopic analysis.

Finally, our study also presents a case for the development and use of live-cell super-resolution microscopy, delivering information on the natural and dynamically evolving state of the biological system free of concerns over fixation artifacts of whatever provenance.

**Table 1:** PFA fixation-induced effects on brain tissue microstructure

Time of fixation Structure	30 min	90 min	>90 min
Extracellular space	No change in VF	No change in VF No change in width	NE (dye uptake)
Global tissue	No visible changes	Cell blebbing	Membrane permeabilization
Astrocytes	NE	No changes in areas of cell bodies and main branches No changes in widths of fine processes	NE
Dendrites Dendritic spines	NE	“Dendritic vacuoles” No changes in spine neck length Decreased spine head area Increased spine neck width	NE

Summary of observed changes in various tissue structures (ECS, astrocytes and dendrites) for different fixation times. VF: volume fraction; NE: not examined.

## Synthesis

Reviewing Editor: Miriam Goodman, Stanford University Decisions are customarily a result of the Reviewing Editor and the peer reviewers coming together and discussing their recommendations until a consensus is reached. When revisions are invited, a fact-based synthesis statement explaining their decision and outlining what is needed to prepare a revision will be listed below. The following reviewer(s) agreed to reveal their identity: Kate O’Neill. Note: If this manuscript was transferred from JNeurosci and a decision was made to accept the manuscript without peer review, a brief statement to this effect will instead be what is listed below.

This manuscript by Idziak et al. characterizes how chemical fixation may introduce artifacts to murine neural tissue visualized by high-resolution (STED) imaging. The effects are seen to be mild during PFA fixation treatments of 30 minutes of less but become more severe after 90 minutes or longer. The results show that immersing the slices in paraformaldehyde does not change the amount of extracellular space. However, there are subtle changes in spine shape with a decrease in the width of the spine neck. Reviewers agreed that this is an important and timely contribution, providing useful information about how a commonly used fixation method, such as buffered formaldehyde, has an impact on the structure of cultured slices. Its impact may well improve experimental design for experiments relying on fixation to analyze brain slice anatomy. Overall, the paper is well written and the results are illustrated with clearly laid out sets of figures. Both reviewers noted that the study will be informative to many groups in the larger scientific community.

Despite the clarity of explanation and thoroughness of the illustrations, however, both reviewers provided suggestions for improvement that we ask you to address. Additionally, Rev2 asks that you be more precise in your discussion of the results and to consider narrowing the scope of writing describing the study. Specifically, although samples analyzed in this study are organotypic slices subjected to buffered formaldehyde (PFA) fixation, the manuscript compares the present results to those observed in other samples like adult brain tissue fixed by perfusion using other fixatives. Such comparisons glide over other factors that may contribute to the differences in the preservation of extracellular spaces, spines, or dendrites. Such factors include, differences in the anatomy of immature brain slices maintained in culture vs. adult brains developing in vivo, effects of fixation by perfusion vs. immersion, and the use of different fixatives. Additional data using other samples (such as acute slices) or other fixatives are welcome. However, such data are not required for the revision. We do ask that the manuscript be revised to use more precise writing in the abstract, introduction and discussion and in the title, which claims findings relevant to all brain tissues.
